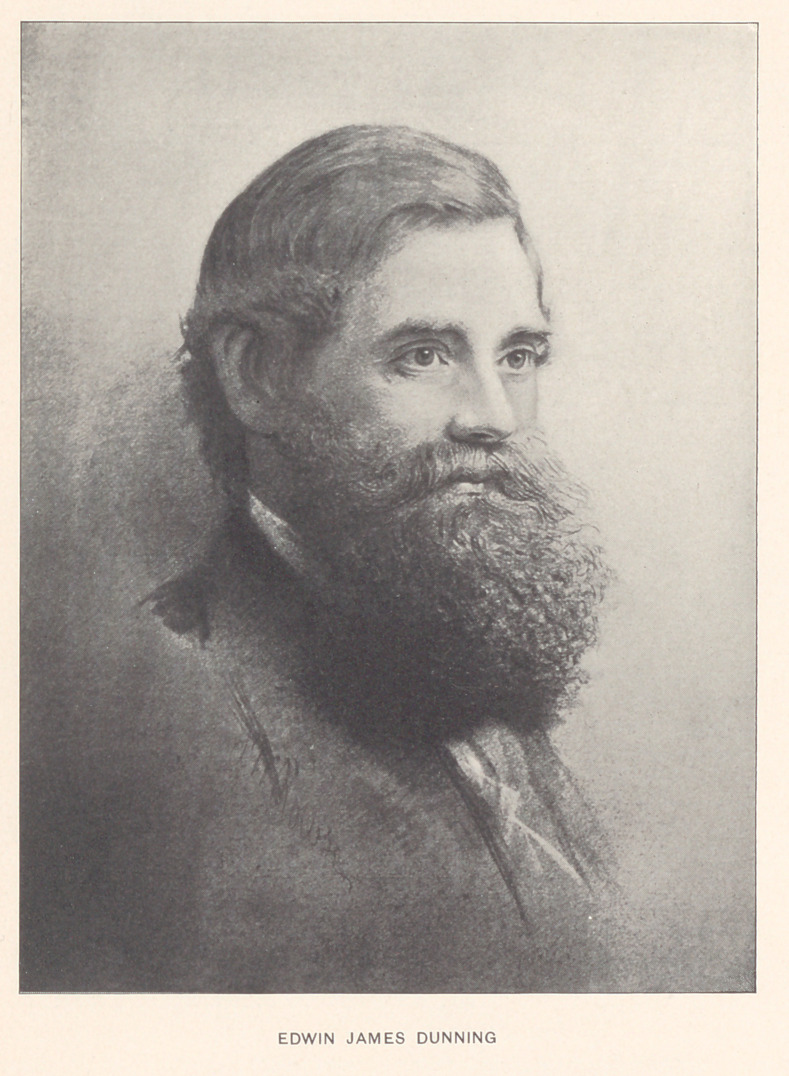# A Biographical Sketch of Edwin James Dunning

**Published:** 1901-11

**Authors:** Charles Otis Kimball


EDWIN JAMES DUNNING

### Biographical Sketch.

### A BIOGRAPHICAL SKETCH OF EDWIN JAMES DUNNING.

BY CHARLES OTIS KIMBALL, M.D.

Dr. Dunning was born in Camillus, N. Y., on July 19, 1821. He was the son of Uriah H. Dunning, a country physician, who also practised dentistry, and Emily James, the sister of Edwin James, physician and botanist, one of the early explorers of the West. He received his early education mostly at Saratoga Springs and Ithaca, where in 1838, at the age of seventeen, with but a few preliminary hints, he began to fill teeth. In the spring of 1839 he entered the office of Dr. Westcott, of Syracuse, studying dentistry with him. In 1844 he came to New York and entered the office of Dr. Eleazer Parmly as an assistant, remaining with him for twelve years. In 1856 he opened an office at 11 Waverly Place, remaining there until he retired from practice in 1874. In 1862 he went to the seat of war as assistant to the Sanitary Commission. During the session of 1867-68 he occupied the chair of operative dentistry in the New York College of Dentistry. In 1872 he went to California, remaining till the spring of 1874, when inflammation in one of his eyes brought on partial blindness and compelled his retirement from practice in May, 1874. He spent the next three years at Lenox, and then went again to California, becoming totally blind that same fall. He remained there till October, 1880. Returning East, he lived at Newton Centre, Elmira, N. Y., and Cambridge, Mass., where he died March 17, 1901, in his eightieth year.

He was married three times,—in 1842 to Lucy Sage, by whom he had six children, the three younger of whom died of diphtheria within two weeks in 1862; in 1869 to Esther Hazard; in 1881 to Christine Boughton. His widow and two sons survive him.

Such is the record of a long life, divided into two periods, which are marked by strong contrast in all outward circumstances. Is there not running through it, now in the sunshine and now in the shadow, a golden thread of purpose which justifies us in pausing for a moment to consider it? Let us study this life, so deeply furrowed by the divine ploughshare, and note its answer to the question.

A country boy of good ancestry, inheriting a somewhat delicate body, a clear mind, and a resolute will, with tastes and aspirations for art, literature, and music, while being trained at a private school for a liberal education was suddenly stopped and at seventeen years of age forced to work out his own career. He began by filling teeth for his fellow-students, then he taught a district school for a winter, and in the spring entered the office of Dr. Westcott, of Syracuse, to take up the study of what now seemed to him likely to be his life work.

At once "ideals of excellence began to form;" he eagerly "caught every hint in matters of practice;" he "learned from every example of good work and from every dentist with whom he conversed." He "dreamed of excelling and being at some time a practitioner of established position in some city," and, as he simply says, "all I can tell about it is that my dream came to pass."

In 1844, at the age of twenty-three years, he came to this city, entering the office of "that excellent and most courteous gentleman," Dr. Eleazer Parmly, then readily acknowledged as at the head of his profession in this city. Here the country boy, under his preceptor's example and teaching, acting upon a fine and delicate native instinct, quickly won a place in the front rank of the profession, which he held for thirty years. Meanwhile, conscious of his inherited delicacy of constitution, he had systematically striven by various means to develop himself, carrying out the same "ideals of excellence" in his physical life as in his higher life work, till he attained a splendid and lasting bodily strength.

He was a most attractive man, in person tall, strong, and vigorous, with a full, low-pitched, well-modulated voice, with a bright eye and a winning smile, careful and exact in speech, polite and courteous in manner, though he could be upon provocation abrupt to sternness. Thorough, painstaking, and quick in his work, unsparing of himself (and his patients where necessary), but always thoughtful and considerate of others, his constant aim was to excel in work, not for his personal gratification but for the good of others, believing that he was working out his Christian life in his daily work at the chair. As his practice grew and his fees increased, each increase meant not so much more added to his income as so much more time and care given to each operation.

In the height of his career, at great personal sacrifice, he accepted the chair of Operative Dentistry in the New York College of Dentistry, and the writer, then a young student, well remembers the address delivered by Dr. Dunning when he assumed the chair. Our profession had seemed to him till then a good enough way of earning a living, though rather uninteresting, but Dr. Dunning's intense enthusiasm, his high point of view, and his appeal to the students to take up their work in the spirit of loving service to humanity, as part of the work to which they were divinely called, touched a chord which has never ceased vibrating, and which has contributed not a little to whatever zeal and faithfulness he may possess. Dr. Dunning's methods, however, were too exacting, his views too radical, and his demands upon the students too severe to please the easy-going crowd, and after a year he resigned his chair, being constrained to by lack of support and encouragement. During the thirty years of marked success in life in all its phases the dominant notes were struck by his enthusiasm, his singleness of purpose, the way in which he threw himself unreservedly into all that he did, doing it heartily as unto the Lord. This gave him a great influence over the young men whom he trained, who bear testimony to his ability and faithfulness by careful attention to the details of their work.

Outside of his work all that is best in life appealed to him; he was fond of art; a member of the Academy of Design, associating much with artists; of music, singing himself; of books and literature, conversing well on many topics.

He loved the country, and turned eagerly to it whenever possible. Riding, driving, walking even for great distances, refreshed and strengthened him. His vacations were passed in the Adirondacks, in the celebrated "Stillman Camp," with Emerson, Lowell, Agassiz, etc.; at Lake George with the artist Durand; in Switzerland and Italy, spending much of his time mountain climbing, of which he was passionately fond, or in taking long walks.

To such a man in the fulness of life, only fifty-three years old, with the promise of years of usefulness before him, came the withering touch upon his eyes, and his professional work was forever laid aside, and for one-third of his life he walked in darkness.

There are two touchstones of character, prosperity and adversity, and we cannot feel sure of any man until we note his reaction to each. We have seen Dr. Dunning in prosperity, everything open to his hand, and that in it all a high purpose and resolute self-devotion to his ideals dominated his life. What did adversity reveal? Let me quote the words of an observer: "After a busy and successful professional career he had suddenly been stricken with blindness, but as we walked up and down the beach at Santa Barbara he talked not of the life that was behind him, but of the life that was before him. He was as one that put on the armor, not as one that was putting it off; he was preparing for life; the life of action had passed, the life of meditation had begun; with eager enthusiasm he talked of the great poets who were to be his companions." They had been the amusement and recreation of his hours of rest, now they were to be his friends and teachers. With cheerful courage he turned his face towards the life of the mind, saying, "Now that I can never read Browning's 'Saul' again, I will commit it to memory." Day by day he committed to memory poems of the great English authors, Wordsworth, Tennyson, Browning, Shelley, till he had over sixty poems stored away in his mind ready for a moment's use.

Then he began the study of Shakespeare's sonnets, memorizing them until he had every one of the hundred and fifty-four at his tongue's end, studying also his other poems, memorizing some, reading and re-reading each play from one to six times, and then meditating upon them "during his waking hours by night and by day through many years," until their meaning came clearly to him as a revelation of the great poet's deepest inner experiences in his poetic life, and he thought with such a spiritual exaltation that his own blindness seemed almost a providential boon. Thus "for more than twenty years Dr. Dunning exhibited to all who knew him the power of the spirit to triumph over bodily infirmities; his years of blindness were years of intellectual growth."

The results of this profound study, this daily living with the poet, were published in 1897, when he was seventy-six years old, under the title "The Genesis of Shakespeare's Art."

These years of darkness were not years of gloom; they were years, as we have seen, of a growing intellectual life. They were filled with intense interest in the life of the world, with prevailing cheerfulness and a serene Christian faith that made them, as he often said, his best years.

And the golden thread running through all, through the shadow and the sunshine, the bright days and the dark weaving both into a beautiful life, making its study worth our while, was the steadfast purpose to regard every power and ability as a divine trust to be used to the full in the service of man.

> "Then soul, live thou upon thy servant's loss,
> And let that pine to aggravate thy store;
> Buy terms divine in selling hours of dross,
> Within be fed, without be rich no more.
> So shalt thou feed on Death; that feeds on men;
> And Death once dead, there's no more dying then."

## Figures and Tables

**Figure f1:**